# UDP-glycosyltransferase genes in trypanosomatid genomes have diversified independently to meet the distinct developmental needs of parasite adaptations

**DOI:** 10.1186/s12862-018-1149-6

**Published:** 2018-03-14

**Authors:** Sara Silva Pereira, Andrew P. Jackson

**Affiliations:** Department of Infection Biology, Institute of Infection and Global Health, University of Liverpool, Liverpool Science Park Ic2, 146 Brownlow Hill, Liverpool, L3 5RF UK

**Keywords:** UDP-glycosyltransferases, Trypanosomatids, Glycosylation

## Abstract

**Background:**

Trypanosomatid parasites such as *Trypanosoma* spp. and *Leishmania* spp. are a major source of infectious disease in humans and domestic animals worldwide. Fundamental to the host-parasite interactions of these potent pathogens are their cell surfaces, which are highly decorated with glycosylated proteins and other macromolecules. Trypanosomatid genomes contain large multi-copy gene families encoding UDP-dependent glycosyltransferases (UGTs), the primary role of which is cell-surface decoration. Here we report a phylogenetic analysis of UGTs from diverse trypanosomatid genomes, the aim of which was to understand the origin and evolution of their diversity.

**Results:**

By combining phylogenetics with analyses of recombination, and selection, we compared UGT repertoire, genomic context and sequence evolution across 19 trypanosomatids. We identified a UGT lineage present in stercorarian trypanosomes and a free-living kinetoplastid *Bodo saltans* that likely represents the ancestral state of this gene family. The phylogeny of parasite-specific genes shows that UGTs repertoire in *Leishmaniinae* and salivarian trypanosomes has expanded independently and with distinct evolutionary dynamics. In the former, the ancestral UGT repertoire was organised in a tandem array from which sporadic transpositions to telomeric regions occurred, allowing expansion most likely through telomeric exchange. In the latter, the ancestral UGT repertoire was comprised of seven subtelomeric lineages, two of which have greatly expanded potentially by gene transposition between these dynamic regions of the genome.

**Conclusions:**

The phylogeny of UGTs confirms that they represent a substantial parasite-specific innovation, which has diversified independently in the distinct trypanosomatid lineages. Nonetheless, developmental regulation has been a strong driver of UGTs diversification in both African trypanosomes and *Leishmania*.

**Electronic supplementary material:**

The online version of this article (10.1186/s12862-018-1149-6) contains supplementary material, which is available to authorized users.

## Background

Trypanosomatid parasites are the causes of several neglected tropical diseases worldwide that put 500 million people and over 60 million cattle at risk of infection [1]. Trypanosomatids include *Leishmania* spp., which cause various kinds of leishmaniasis; stercorarian trypanosomes such as *Trypanosoma cruzi*, the cause of Chagas disease in central and south America; and salivarian trypanosomes such as *Trypanosoma brucei*, the cause of African trypanosomiasis in humans and animals, (as well as *T. vivax* and *T. congolense* that cause disease exclusively in animals). Collectively, these vector-borne diseases have a significant impact on human and animal health, and are a profound constraint on the socio-economic development of low and middle-income countries.

The life cycles of trypanosomatids may be monoxenic or dixenic. All human and animal parasites are dixenic, cycling between a vertebrate host and an invertebrate vector. African trypanosomes alternate between several life forms, procyclic, epimastigote and metacyclic stages in the tsetse fly (*Glossina* spp.) and extracellular bloodstream-forms in the mammalian host. *T. cruzi* infects a wide range of mammals and is transmitted by the bite of triatomine bugs. *Leishmania* spp. alternate between a motile, promastigote form in a sand-fly vector, and an intracellular amastigote form in their mammalian host. Besides these, and many other dixenic parasites, there are multiple genera of monoxenic trypanosomatids that parasitize insects and are transmitted through the faecal-oral route, such as *Crithidia*, *Leptomonas* and *Lotmaria* [2–4]. Regardless of whether they have one or multiple hosts, all trypanosomatids have a complex development and are able to adopt multiple cell morphologies depending on the precise host environment they inhabit [5, 6]. Associated with these different cell morphologies, are characteristic cell-surface architectures that are typically parasite-specific and substituted during transmission between hosts [7–9].

The surface of trypanosomatids is composed of several macromolecules, some of which are subject to glycosylation, for example, through the addition of a glycophosphatidylinositol (GPI) anchor [10]. UDP-glycosyltransferases (UGTs) catalyse the transfer of N-acetylglucosamine (GlcNAc) residues from UDP-GlcNAc to phosphatidylinositol [11, 12] in the first step of GPI anchor synthesis, but also play a crucial role in the synthesis of glycans of various functions, contributing to the extraordinary collection of glycoconjugates that decorate the surface of trypanosomatids [13].

UGTs are part of a superfamily of glycosyltransferases (GT) present in all organisms, which typically play a role in detoxification and homeostatic processes [14]. Three types of GTs have been characterised (A-C): GT-A share a catalytic domain, the DXD motif, whose carboxylated side chains coordinate enzymatic activity; GT-B are very diverse; and GT-C have only recently been described from iterative sequence searches with a single 3-D structure not supporting the presence of a common active site [15]. Trypanosomatid UGTs belong to the inverting GT-A family 31 (GT31 in CAZY nomenclature), a family present in eukaryotes and prokaryotes. In plants, GT31 includes enzymes involved in proteoglycan and N-glycan synthesis [16]; in mammals it includes chondroitin synthases, responsible for the synthesis of glycosaminoglycan chains that regulate homeostatic processes, such as cell proliferation and extracellular matrix deposition [17], and Fringe proteins which modulate the Notch signalling pathway [18]. In bacteria, GT31 enzymes also play an important role in epitope synthesis, such as the catalysis of the final steps in formation of the *O* antigen repeating unit in pathogenic *E. coli*, through the glycosylation of the nonreducing end of oligosaccharides [19]. In trypanosomatids, this family has expanded greatly compared to other eukaryotes and its function closely relates to surface decoration [13]. In these organisms, UGTs can accept several sugar nucleotides, some of which are common to all three groups (i.e. GDP-α-D-mannose, UDP-α-D-*N*-acetylglucosamine, UDP-α-D-glucose, UDP-α-galactopyranose, and GDP-ß-L-fucose), while others are specific to one or two organism families (e.g UDP-α-D-xylose, UDP-α-D-glucuronic acid are found exclusively in *T. cruzi*) [20]. Despite this wide variety of substrates, for the purpose of this study, we are focusing on those enzymes related to galactose and/or those directly involved in the synthesis of glycosydic conjugates.

In *Leishmania*, UGTs are essential for making the phosphosaccharide repeats [PO_4_-Man-Gal] that compose the parasite dense glycocalyx, using UDP-galactose as the glycosyl donor [21]. Simultaneously, a subset of UGTs belonging to the side chain galactose-related gene families (SCG, SCGL, SCGR) catalyse the attachment of Gal(ß1,3) side chains to the phosphoglycan (PG) polymer repeating units of the lipophosphoglycan (LPG) coat. The PG repeats are required for parasite survival in the sandfly midgut, where parasite differentiation to the replicating procyclic promastigote stage occurs [22]. Whilst most microbial adhesins are proteins that interact with various molecules in host epithelial receptors, *Leishmania papatasi* stage-specific adhesion potential is provided by LPG, a glycoconjugate interacting with lectin receptors in the epithelium of the sandfly midgut [23]. The galactose side chains permit binding and adhesion to lectins in the midgut epithelium during the digestion process, so the parasite can avoid excretion with the peritrophic matrix [24].

In African trypanosomes, UGTs are involved in the synthesis of complex poly-N-acetyllactosamie-containing type N-linked and GPI-linked glycans. N-linked glycans can have various functions: on VSG, they are predicted to assist the protection of invariant surface antigens by filling the spaces between VSGs [25]; on the transferrin receptor, they ensure enough space is left at the flagellar pocket to allow efficient binding of the receptor to transferrin [26]; and on the lysosome-associated membrane protein p67, N-linked glycans might function as internalisation signals for endocytosis [27]. GPI-linked glycans of procyclins play a role in tsetse fly colonisation and, in the mammal, as VSG GPI-anchor side chains [28, 29]. Since UDP-Gal-dependent glycosylation pathways are essential for the survival of *T. brucei* in both insect and mammal forms [30, 31], UGTs make logical targets to understand parasite-host interactions.

The publication of genomes for most trypanosomatid species [2–4, 32–39] together with transcriptomic and proteomic studies [40–44] demonstrated that trypanosomatids possess large repertoires of UGT isoforms encoded by multi-gene families often found in irregular tandem gene arrays. The recent publication of a genome sequence for the free-living kinetoplastid *Bodo saltans* [45] provides an out-group for a comparative analysis of trypanosomatid UGT genes, able to answer fundamental questions about their diversity.

Three main reasons make UGTs sensible study targets: i) Despite being a multi-copy gene family with distinct repertoires across species and important roles in pathogenesis, their diversity across the genus is poorly understood; ii) The understanding of its diversity may elucidate phenotypic differences in disease progression; and iii) Through genomic comparison we can identify shared and species-specific loci, as well as stage-specific isoforms, to expedite the search for suitable drug and transmission targets.

Here we describe the phylogeny and comparative genomics of UGT genes in trypanosomatids and *Bodo saltans* with particular emphasis on African trypanosomes and *Leishmania*. We aim to identify monophyletic free-living (*B. saltans)* and parasitic (trypanosomatid) UGTs to understand more about their ancestral form and the origin of family expansion. In this process, we investigate orthology across parasites to know whether UGT expansion was independent in distinct parasites, and understand the role of recombination among paralogs and of selection in gene divergence. Finally, we interpret those results in the context of available gene expression and functional studies, whilst searching for evidence of functional differentiation, since non-redundant paralogs under strong negative selection could offer targets for functional studies and interventions.

## Methods

### Data collection and nomenclature

Annotated UGT sequences were obtained from genome sequences of *Trypanosoma cruzi* CL Brenner Esmeraldo-like, *T. rangeli* SC58, *T. grayi* ANR4, *T. brucei* TREU927, *T. congolense* IL3000, *T. vivax* Y486, *Leishmania major* Friedlin, *L. infantum* JPCM5, *L. mexicana* MHOM/GT/2001/U1103, *L. tarantolae* Parrot-Tarll, *L. enriettii* LEM3045, *L. braziliensis* MHOM/BR/75/M2904, *Leptomonas pyrrhocoris* H10, and *Crithidia fasciculata* Cf-Cl hosted by TritrypDB v.28 (http://tritrypdb.org) [46]; *Bodo saltans* hosted by the GeneDB website (http://genedb.org) [47]*;* and *Angomonas deanei and Strigomonas culicis* hosted by Ensembl Protists v.31 (http://protists.ensembl.org). Additionally, a sequence similarity search with tBLASTn using *T. brucei*, *L. major* and *B. saltans* UGTs as query was performed to identify relevant genes annotated as hypothetical.

To expand the sample repertoire of monoxenic species, the genome sequences from *Crithidia acanthocephali* and *Lotmaria passim* unannotated genomes were retrieved from NCBI (http://www.ncbi.nlm.nih.gov/genome). These were inspected for UGTs by sequence similarity search with tBLASTn using its closest relative, *C. fasciculata*, UGTs as the query. Identified putative UGTs were named *L. passim*1–4 and C. *acanthocephali*1–4.

UGT Sequences from *Trypanosoma gambiense* DAL972 and *Trypanosoma evansi* (hosted by TritrypDB v.28 (http://tritrypdb.org) [46]), and from *Trypanosoma equiperdum* (hosted by NCBI (http://www.ncbi.nlm.nih.gov/genome)) were also inspected. However, as they present the same repertoire as *T. brucei* TREU 927, the latter was used as a representative of the Trypanozoon subgenus.

The presence of the conserved UDP catalytic domain previously described (DXD) in the sequences was a requirement for the inclusion in this study [48].

### Multiple sequence alignment

Translated nucleotide sequences were aligned with ClustalW [49] using BioEdit 7.2.5 (http://www.mbio.ncsu.edu/BioEdit/bioedit.html) and back translated, producing a nucleotide alignment of 1005 nucleotides. Subsequently, aligned nucleotide sequences were translated again, resulting in a protein alignment of 335 amino acids around the catalytic domain, after trimming non-conserved regions. This corresponded to 23–93% of the full glycosyltransferase proteins sequence, due to *Leishmania* spp. having a large specific insertion. When analyzed separately, the African trypanosome alignment was 305 amino acids long, while the *Leishmaniinae* alignment was 824 amino acids long.

### Phylogenetic estimation

The UGT phylogeny was estimated from protein sequence alignments with maximum likelihood (ML) under a WAG+Γ substitution model [50] using PHYML v3.0 [51] and under a VT + F + R6 substitution model [52] using IQTree [53]. Robustness was assessed with 500 bootstrap replicates. We also attempted to estimate a phylogeny using Bayesian inference (BI) but the analysis failed to converge on stable parameter values and therefore was not pursued.

The UGT phylogenies of African trypanosomes and *Leishmaniinae* were estimated from nucleotide sequence alignments with ML using PHYML v3.0 [51], BI using MrBayes v3.1.2. [54, 55], and Neighbor-Joining (NJ) using MEGA7 [56], and from protein sequence alignments with three methods: ML using PHYML v3.0 [51], BI using MrBayes v3.1.2. [54, 55], and ML using IQTree [53].

Optimal substitution models for PHYML amino acid trees were found with the Smart Model Selection option in PHYML, using the Akaike Information Criterion (AIC_c_). PHYML protein trees were estimated with WAG+Γ (African trypanosomes) [50] or LG + Γ model (*Leishmaniinae*) [57]. Optimal substitution models for IQTree ML trees were found with the built-in ModelFinder tool [58]. IQTree protein trees were estimated with WAG+G4 model (African trypanosomes) [50] or VT + F + R4 model (*Leishmaniinae*) [52]. Nucleotide trees were estimated with the GTR+Γ model [59] with 500 bootstrap replicates.

The BI trees were estimated with gamma rates function in MrBayes and four Markov chain Monte Carlo chains run in parallel over 2,500,000 generations, with a burnin of 5000. The nucleotide BI trees were estimated with default parameters whereas the protein BI trees were estimated with a fixed WAG+Γ model. Posterior probabilities of each node were used to assess accuracy of BI trees.

Nucleotide NJ trees were estimated using logdet genetic distances to correct base composition bias [60] using MEGA7 [56] and robustness was assessed with 500 bootstrap replicates.

### Tests for recombination

Evidence for recombination was investigated in *L. major*, *L. infantum*, *L. mexicana*, *T. brucei*, and *T. congolense*. For *Leishmania*, SCG and SCGR subfamilies were separately analyzed. For African trypanosomes, each lineage was separately analyzed. Four sequences were randomly selected for each species and subject to different tests. In *Leishmania*, a negative control comprised of four sequences known not to recombine (one SCG, one SCGR, one SCGL, and one SCGR gene phylogenetically closer to SCGL) were included. In African trypanosomes, the negative control was comprised of all genes from lineages 2–5.

Recombination probability was detected with the pair-wise homoplasy index (PHI) [61] as part of the SplitsTree package [62]. Breakpoints were predicted with the Genetic Algorithm for Recombination Detection (GARD) [63], run using the REV model, under the AIC_c_ information criterion. The KH test was applied to test for rate heterogeneity to prevent false positives arising from significant topological incongruences rather than recombination. These tests informed on the likelihood of recombination affecting sequence evolution. The breakpoint(s) identified with GARD were used to split the sequences into non-recombinant parts before subsequent analyses of selection to prevent false positives due to recombination.

### Positive selection tests

To evaluate whether positive selection was affecting sequence evolution, full sequences where recombination was unlikely and non-recombinant partial sequences were subject to six site-level selection tests: Single Likelihood Ancestor Counting (SLAC) to perform ancestral reconstruction; Fixed Effects Likelihood (FEL) to directly estimate *dN*/*dS* ratios [64]; Random Effects Likelihood (REL) to infer selection pressures using an empirical Bayes approach and model *dN*/*dS* ratios at individual sites based on a pre-defined distribution; Partitioning Approach for Robust Inference of Selection (PARRIS) [65] to test for alignment-wide evidence of selection taking into account recombination and synonymous rate variation; Fast Unbiased Bayesian Approximation (FUBAR) to estimate the *dN*/*dS* ratio based on Bayesian Inference using a MCMC routine [66]; and the standalone package Phylogenetic Analysis Using Maximum Likelihood (PAMLx) to construct likelihood ratio tests [67].

Significance thresholds for recombination were *p*-value < 0.05 and posterior probability > 0.9. For sites to be considered under positive selection, support by 4 out of 5 tests was required. Unless specified, all programs were hosted at the DataMonkey server (http://datamonkey.org).

## Results

**Fig. 1:**
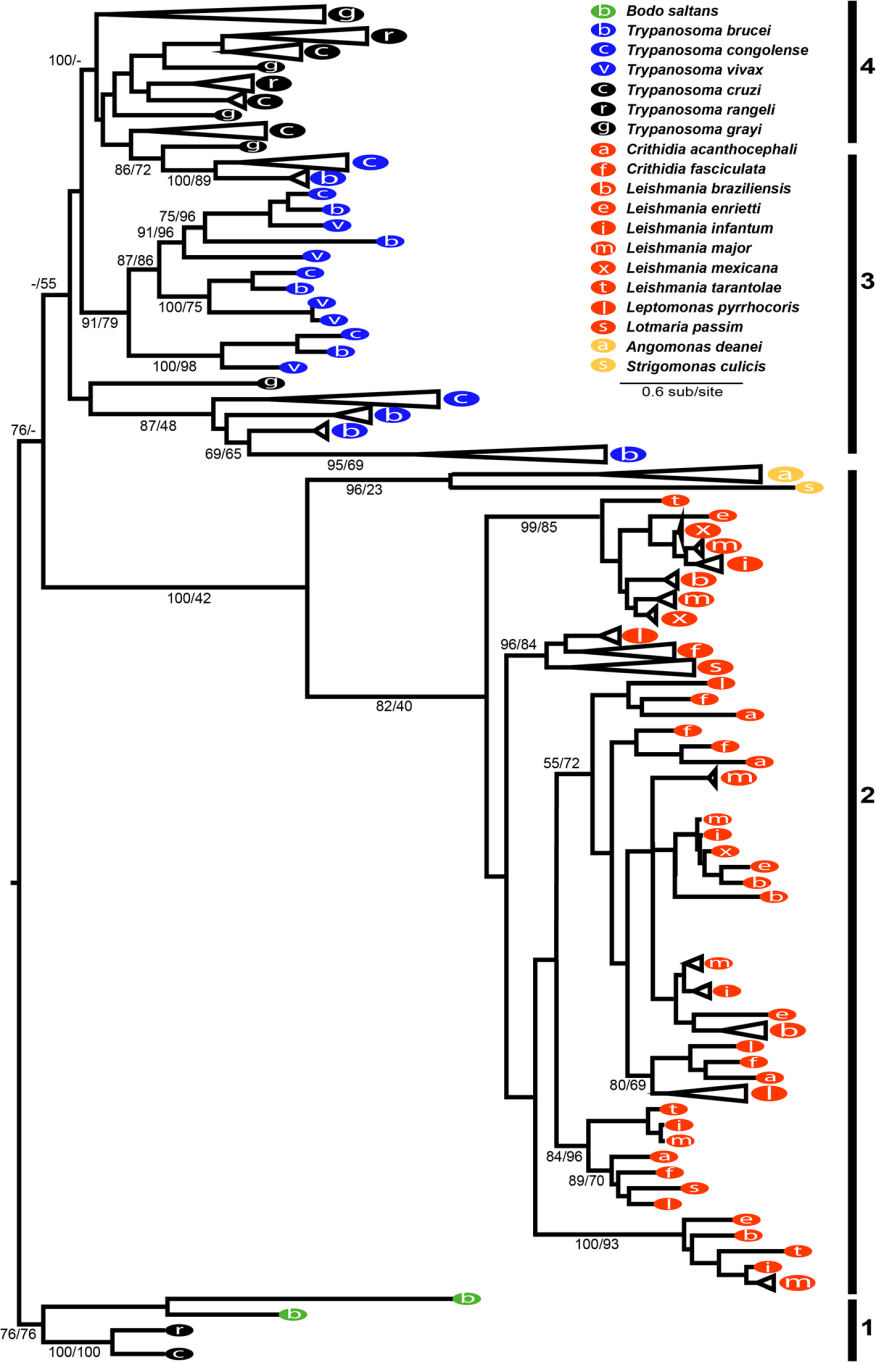
Consensus maximum likelihood phylogeny of UDP-glycosyltransferases protein sequences from diverse trypanosomatids and *B. saltans*. The phylogeny was estimated with PHYML using a maximum likelihood method with a WAG+Γ model and 500 bootstrap replicates. Terminal nodes are named with Genedb [47] and Tritrypdb [46] identifiers; internal nodes are labeled with bootstrap percentages for maximum likelihood estimated with PHYML [51] and IQTree [53]. Tips are labeled according to key. The tree is rooted with two *Bodo saltans* sequences as the outgroup. Refer to text for labels 1 to 4

We estimated a UGT maximum likelihood phylogeny (Fig. 1) from a 335 residue multiple alignment of 236 protein sequences. Among these sequences were 2 UGT sequences from the non-parasitic out-group *B. saltans*, 2 from *Angomonas deanei*, 1 from *Strigomonas culicis*, 82 from stercorarian trypanosomes (3 species), 71 from African trypanosomes (3 species), and 70 from *Leishmaniinae* (10 species). It is immediately clear from this phylogeny that trypanosomatids have greatly expanded their UGT repertoires relative to the free-living *B. saltans* (e.g. ratios of 1:13 for *T. brucei*; 2:13 for *L. major*; 1:23 for *T. cruzi*). Furthermore, UGT repertoires have been elaborated to different extents in trypanosomatid genera, e.g. *T. brucei*: *L. major* ratio of 2:1).

The tree topology broadly reflects the major trypanosomatid lineages and contains four main features, (numbered 1–4 in Fig. 1), that will be examined further: a clade comprising *B. saltans* sequences and rare orthologs from stercorarian species (‘the ancestral lineage’) (1); a clade of *Leishmaniinae* sequences (2); two clades of African trypanosomes (3). Most stercorarian trypanosome sequences clustered together (although some *T. grayi* sequences were ambiguous) but without adequate node support (4). The lack of species diversity hampers orthology analysis and thus we have not examined stercorarian sequences further in this study.

### An ancestral UGT lineage shared by stercorarian trypanosomes and *B. saltans*

The ancestral lineage is composed of four genes retaining orthology: two *B. saltans* (BSAL_27930 and BSAL_69925), one *T. cruzi* (TcCLB.503487.50), and one *T. rangeli* (TRSC58_00816), all close in length (352 to 495 amino acids). The *B. saltans* sequences share 31% overall identity between each other and 34–36% identity with *T. cruzi*, *T. rangeli* and *T. grayi* (Tgr.1587.1000). The latter was not included in the phylogeny due to its short length (90 amino acids). The absence of this lineage of UGTs in *Leishmaniinae* and African trypanosomes suggests post-speciation gene loss. Transcriptomic data from genomic microarrays show TcCLB.503487.50 is constitutively expressed, being the most abundant in amastigotes and the least in epimastigotes [68]. The genomic locus of these genes could not be investigated due to the current quality of the assemblies of *T. rangeli* and *T. grayi* genomes.

A search for similar sequences in *Euglena gracilis* transcriptome [69] and *Trypanoplasma borreli* [45], *Phytomonas* sp. isolates EM1 and Hart1 [70], *Paratrypanosoma confusum* [71] and *Naegleria fowleri* [72] genomes did not produce any relevant matches.

### *Leishmania* UGT repertoire derives from ancestral tandem array

**Fig. 2:**
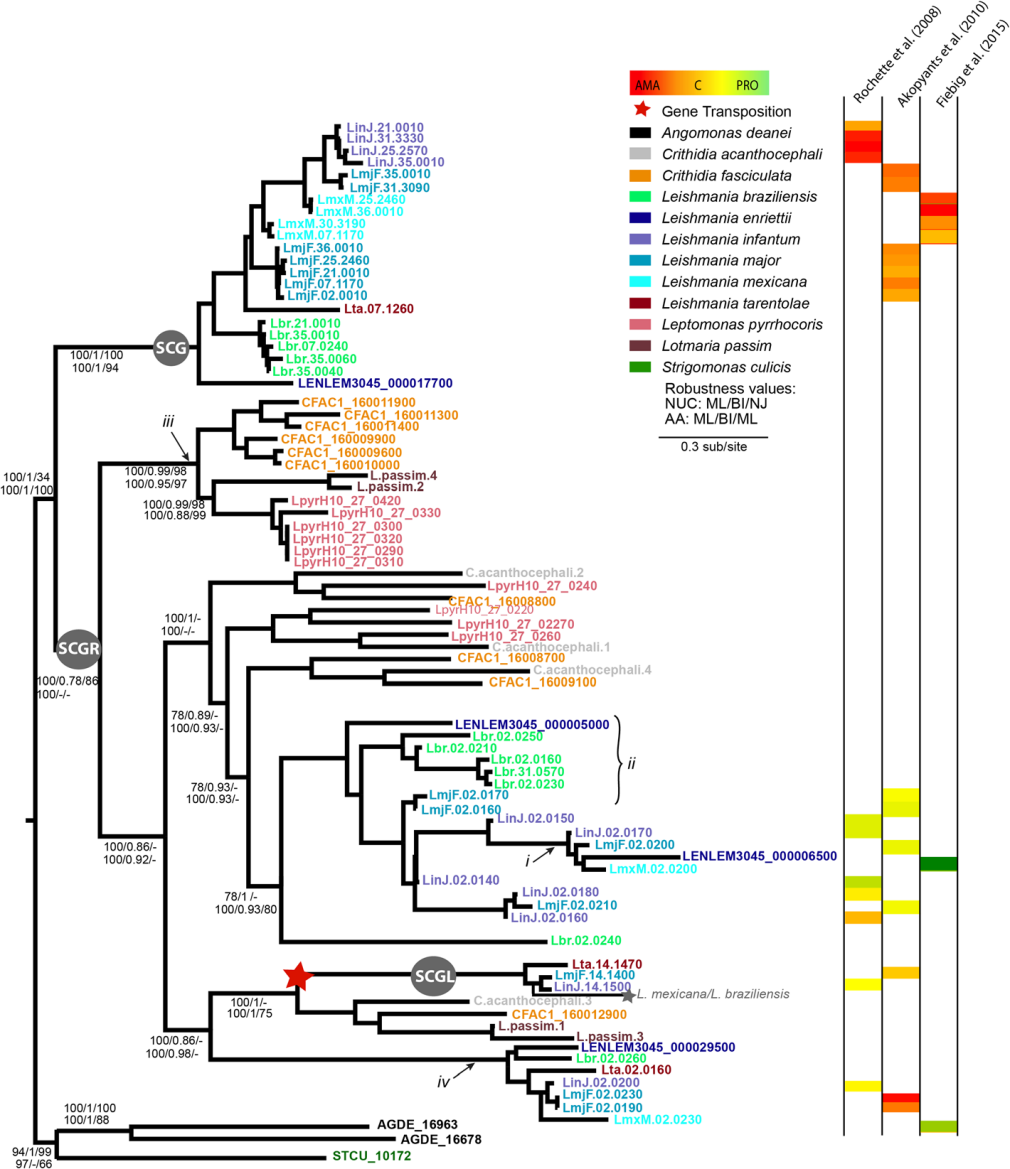
Consensus maximum likelihood phylogeny of UDP-glycosyltransferases nucleotide sequences from *Leishmaniinae*. The phylogeny was estimated with PHYML with a GTR+Γ model and 500 bootstrap replicates. Terminal nodes are named with Tritrypdb [46] identifiers; internal nodes are labeled with bootstrap percentages for maximum likelihood (ML), posterior probabilities (BI), and logdet (NJ) methods for nucleotides sequences and with bootstrap percentages for ML (PhyML [51]), posterior probabilities (BI), and bootstrap percentages for ML (IQTree [53]) for protein sequences. Dashes represent differences in topology. Tree is rooted with *Angomonas deanei* and *Strigomonas culicis* as outgroup. The gray star indicates a gene loss in *L. mexicana* and *L. braziliensis* after the gene transposition event to chromosome 14. Tips are labeled according to key. Clades are identified as SCG, SCGR, and SCGL according to previous annotation in *L. major* (Dobson et al., 2006). Available expression data is represented as log2 fold change of amastigote (AMA), constitutive (C), or promastigotes (PRO) in a heat map according to study reference. Asterisk indicates data is transcriptomic. Refer to text for labels i to iv

The UGT phylogeny raised specific questions about the different gene lineages in the *Leishmaniinae* subfamily so we investigated it further by building a *Leishmaniinae*-only phylogeny based on a longer multiple sequence alignment (Fig. 2), comparing genomic loci and looking at available gene expression data.

**Fig. 3:**
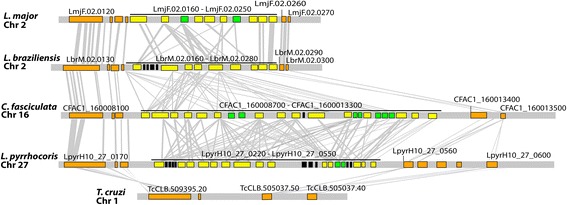
Structural conservation of genomic loci containing UDP-glycosyltransferases among *Leishmaniinae* species. Conserved genomic locus in *L. major* Friedlin, *L. braziliensis* M2904, *Crithidia fasciculata* CfC1, *Leptomonas pyrrhocoris* H10, and *T. cruzi* Esmeraldo. The UDP-glycosyltransferase genes are shaded yellow, flanking genes are shaded orange, arabinosyltransferase genes are shaded in green, and other hypothetical proteins in the array are shaded in black; sequence homology is illustrated by grey vertical bars. Gene terminology is according to Tritrypdb [46] identifiers. Comparisons were obtained with Artemis Comparison Tool (ACT) [85]

All *Leishmaniinae* species have multiple UGT genes organised in a tandem array in chromosome 2 (*L. major*); these are the side chain galactose receptors (SCGR) [48, 73]. The tandem array is located in a conserved genomic locus flanked by a putative phosphatidylinositol kinase related protein (LmjF.02.0120) and small GTP binding protein rab6-like protein (LmjF.02.0260) (Fig. 3). As shown in Fig. 2, SCGR genes cluster by position between closely related species (e.g. LmjF.02.0200 and its neighbors, denoted by “i”), but by species between distant relatives (e.g. Lbr.02.0250 and its relatives, denoted by “ii”). Furthermore, there are examples of extensive gene duplication in one lineage of the monoxenic species (e.g. CFAC1_160011900, denoted by ‘iii’) and in *L. braziliensis* (e.g. Lbr.02.0250). This suggests slow but on-going concerted evolution arising from rapid gene duplication and resulting in the loss of orthology over time. One possible exception to this prevailing pattern is Lbr.02.0260 and its orthologs (denoted by “iv”). Although orthology between *L. major* and *L. braziliensis* is mostly absent within the array, this is an example of a lineage present in all *Leishmania* species (LmjF.02.0230), whose sequences show a unique change in the catalytic domain from DDD to YDD, hinting functional differentiation.

Orthology is also conserved in the *Leishmania*-specific single-copy lineage located in chromosome 14, which has been previously identified in *L. major* as side-chain galactose ligand (SCGL) [73]. The phylogeny suggests it derives from a single transposition event from the array to chromosome 14 in the *Leishmania* ancestor. The absence of a gene at this particular locus in *L. braziliensis* and *L. mexicana* indicates loss in these species (Additional file 1: Figure S1).

Unlike the two previous lineages, the last lineage of UGTs in the *Leishmaniinae* sub-family, which comprises the *Leishmania*-specific side-chain galactose (SCG) genes [73], has a dynamic of concerted evolution. These locate at the subtelomeres of multiple chromosomes, but although the genomic loci are structurally conserved, these genes do not retain orthology between the different species. Additional file 2: Figure S2 shows an example of this at the distal telomere of chromosome 25. This scenario suggests that this gene lineage transposed to telomeres in the *Leishmania* ancestor and has since expanded to other chromosomes perhaps by telomeric exchange, providing strong evidence for concerted evolution.

We have examined existing evidence for protein expression of SCG genes for *L. infantum* [74], *L. major* [40], and *L. mexicana* [75] (Fig. 2). Available microarray data for *L. infantum* [74] reveal three of four SCG genes being differentially expressed in the amastigote stage, as opposed to all SCGR genes being constitutively expressed. The SCGL gene LinJ.14.1500 was not detected in the study. Proteomic analysis in *L. major* showed differential expression at the amastigote stage of LmjF.02.0230 only, but all seven SCG genes and LmjF.02.0190 seem to be more abundant in the amastigote stage (Fig. 3). The remaining SCGR and the SCGL genes do not show developmental regulation [40]. RNAseq data from *L. mexicana* shows also preferential expression of SCG genes in the amastigote stage and of SCGR genes in the promastigote stage [75] (Fig. 2).

In summary, SCG genes seem generally more abundant in the amastigote stage of *Leishmania* species; SCGR generally constitutively expressed; and SCGL present in very low abundances. This suggests that developmental regulation accounts for some degree of gene differentiation.

Prior to selection testing, evidence for recombination was investigated. Both recombination tests suggest *L. major* SCG genes to be under recombination, with GARD identifying one significant breakpoint at nucleotide 489. Trees inferred from GARD were fed into six tests for selection. Only PAMLx and FUBAR found evidence for positive selection, but not significant compared to the negative control. Selection tests for sequences where GARD did not predict significant breakpoints were not consistent, but no sites under positive selection were identified in any of the sequence collections by more than 3 out of 6 tests (Additional file 3: Table S1). Hence, there is little evidence for *Leishmania* UGTs to be under positive selection.

### Seven lineages underline the UGT repertoire in African trypanosomes

**Fig. 4:**
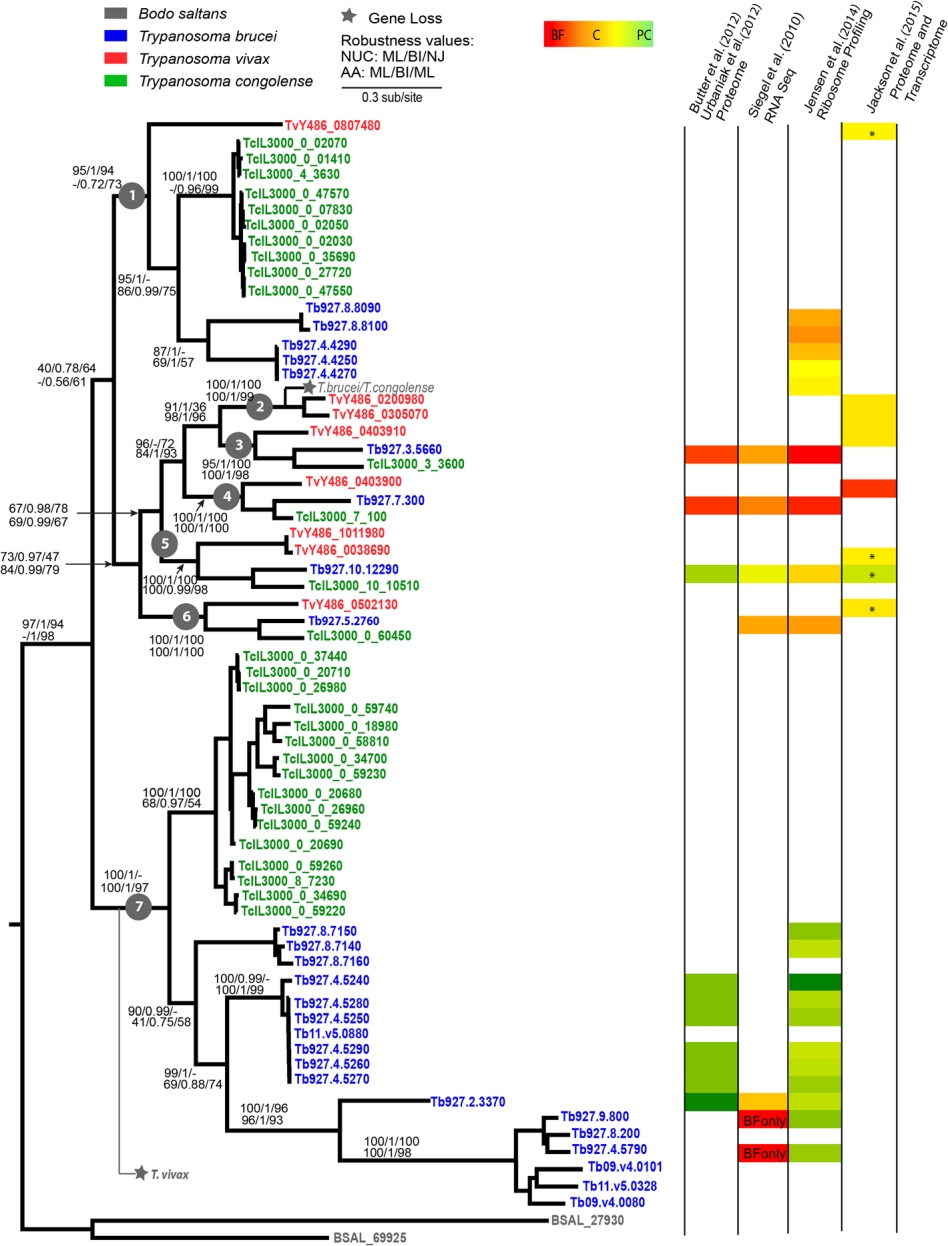
Consensus maximum likelihood phylogeny of UDP-glycosyltransferases nucleotide sequences from African trypanosomes. The phylogeny was estimated with PHYML a GTR+Γ model and 500 bootstrap replicates. Terminal nodes are named with Genedb [47] and Tritrypdb [46] identifiers internal nodes are labeled with bootstrap percentages for maximum likelihood (ML), posterior probabilities (BI), and logdet (NJ) methods for nucleotides sequences and with bootstrap percentages for ML (PhyML [51]), posterior probabilities (BI), and bootstrap percentages for ML (IQTree [53]) for protein sequences. Dashes represent differences in topology. The tree is rooted with *Bodo saltans* as outgroup. Tips are labeled according to key. Available expression data is represented as log2 fold change as procyclic (PF), constitutive (C), or bloodstream form (BF) in a heat map according to study reference. Asterisk indicates data is transcriptomic

To further understand the different lineages of UGTs in African trypanosomes, we estimated a phylogeny of these species, with *B. saltans* as the out-group (Fig. 4). Furthermore, we investigated the genomic loci of the distinct lineages and interpreted them in the context of gene expression.

The phylogeny of UGTs in African trypanosomes shows seven lineages present in the common ancestor (numbered 1–7 in Fig. 4) that retain orthology or co-orthology between species. Lineages 2–6 remain mostly single-copy orthologs. Evidence for conservation of genomic synteny is sporadic due to the quality of current genome assemblies of *T. congolense* and *T. vivax*. For example, in *T. brucei* and *T. congolense*, lineage 4 locus is conserved, being flanked by a leucine-rich repeat protein (Tb927.7.290) and a thioestherase-like superfamily protein (Tb927.7.330), but the *T. vivax* contig containing the former does not span the UGT gene. Similarly, lineage 6 locus seems conserved in all three species, being delimited by a methyltransferase domain containing protein (Tb927.10.12270) and a helicase-like protein (Tb927.10.310), although sequence gaps in *T. congolense* assembly preclude a final decision.

The pattern of orthologs among the seven lineages is disrupted on occasions. Lineage 2 was lost from *T. brucei* and *T. congolense*, while lineage 7 has been lost in *T. vivax*, but vastly expanded in the remaining species. Within *T. congolense* and *T. brucei*, concerted evolution of paralogs occurs, with genes arranged by species in lineages 1 and 7 and conservation of subtelomeric locations, suggesting expansion is arising from transposition of UGTs between these dynamic regions of the genome.

The analysis of the available expression data at the proteomic level reveals some developmental regulation of *T. brucei* genes, with lineages 3 (Tb927.3.5660) and 4 (Tb927.7.300) being differentially expressed in the bloodstream form, and lineages 5 (Tb927.10.12290) and 7 (Tb927.2.3370 and Tb927.4.5240 to Tb927.4.5290) being preferentially expressed in the procyclic form of the life cycle [41, 42].

At the transcriptomic level, the higher abundance in bloodstream forms of Tb927.3.5660 and Tb927.7.300 is already significant, but not of Tb927.10.12290 in procyclics. Transcriptomic data also shows Tb927.5.2760 as differentially regulated in bloodstream forms. Tb927.2.3370 and Tb927.4.5240 seem to be constitutively transcribed, whilst Tb927.9.800 and Tb927.4.5790 are preferentially transcribed in BSF [43].

Available ribosomal profiling studies agree with proteomic data results and suggest higher abundance at the bloodstream form stage of Tb927.8.8090, Tb927.8.8100, Tb927.4.4290, Tb927.4.4250 and Tb927.4.4270 (Jensen et al., 2014) (Fig. 4). Functional characterization of these proteins is yet to be published.

Expression data for *T. congolense* is not available, but the *T. vivax* expression study revealed higher protein abundance of TvY486_0403910, TvY486_0200980 and TvY486_0305070 (corresponding to lineages 2 and 3) in bloodstream forms compared to metacyclics (maximum fold change of 1.42), as well as of TvY486_0403900 (lineage 4) when compared to epimastigotes (maximum fold change of 11.02) [44]. Transcriptomic data suggest differential transcription of TvY486_0403900 between bloodstream forms, epimastigotes and metacyclics (fold change of 2.66 and 3.54, respectively) [44].

In summary, the UGTs repertoire of African trypanosomes seems to be under strong developmental regulation, corroborating the hypothesis of functional differentiation within the family.

To test the contribution of selection to UGT expansion in African trypanosomes, we first searched for evidence of recombination and subsequently performed six tests of site-level selection. Three tests found evidence for recombination among *T. congolense* genes with three significant breakpoints identified by GARD taking into account rate variation. The six tests for positive selection performed did not show evidence for positive selected sites; only PAML identified one site under positive selection at nucleotide 257 of the alignment.

Selection tests for sequences where GARD did not predict significant breakpoints did not find any evidence for positive selection at the site level, but rather negative selection in lineages 2–6, suggesting UGT family expansion is not driven by positive selection or gene conversion.

## Discussion

All trypanosomatids sampled, with the exception of *Angomonas deanei* and *Strigomonas culicis,* have a broad UGT repertoire, which suggests these enzymes play important roles for parasite survival. The lineage present in *B. saltans* and stercorarian trypanosomes may represent a remnant of the ancestral repertoire, which expanded independently in trypanosomes and Leishmaniinae. The trypanosomatid UGT phylogeny lacks support in the stercorarian trypanosomes and *T. grayi* nodes, which could potentially be improved through the introduction of sequences from related trypanosomes, such as *T. theileri* or *T. avium.* These would strengthen robustness of *T. grayi* nodes and help deciphering the relative phylogenetic distance between *T. grayi* UGTs and the remaining trypanosomes.

### The ancestral lineage could reveal the reasons behind parasite-specific UGT innovations

The UGT ancestral lineage retained in *B. saltans* and stercorarian trypanosomes indicates that the UGT repertoire of the ancestral organism was considerably smaller, with fewer loci, supporting the theory that UGT expansion in trypanosomatids is a parasite-specific innovation. UGT expansion is occurring under different dynamics in *Leishmania* spp. and trypanosomes and UGTs are evolving to perform specific, essential roles in the life cycles of these parasites. Comparing the UGT ancestral lineage, which we term the ‘protolog’, in the free-living *B. saltans* with parasite-specific UGTs can be useful to uncover the reasons behind UGT expansion and the benefits gained by these innovations. Comparing ‘the protolog’ with its parasitic homologs can begin to reveal the role of parasite-specific UGTs in the origin of parasitism. At the moment, the phylogeny shows that, in *T. cruzi*, the gene belonging to the ancestral lineage is constitutively transcribed, but more abundant in amastigotes (the intracellular stage in the mammal host), which contrasts with the transcriptomic data available for other UGT genes (TcCLB.511339.30; TcCLB.508673.20; TcCLB.511395.120; TcCLB.508605.20; TcCLB.510553.50; TcCLB.510071.30; TcCLB.504557.20; TcCLB.508975.30), mostly more abundant in trypomastigotes (the bloodstream stage of the parasite) [68].

### UGTs conserved across *Leishmania* probably encode functionally distinct and non-redundant enzymes

Current knowledge of UGTs in *L. major* shows functional differentiation between SCG, SCGR and SCGL sub-families [48, 73]. The phylogeny in Fig. 2 clearly supports that observation for SCG and most SCGR genes, but suggests that LmjF.02.0230 and Lmj.02.0190 (also known as SCGR1 and 4) might be functionally distinct from the remaining SCGR genes as shown by their positioning in a paraphyletic clade with SCGL. SCGR genes are arranged in a tandem array with members of the arabinosyltransferase family. This array is conserved across the *Leishmaniinae* subfamily with striking amino acid conservation, particularly in the surroundings of the “DXD motif” catalytic domain. This domain is conserved across most eukaryotic GT-A proteins, but is modified in SCGR1 and 4 (LmjF.02.0230 and Lmj.02.0190) in all *Leishmania* species (i.e. DDD to YDD), suggesting a parasitic innovation that may result in functional adaptation of these enzymes. When these genes were described in *L. major*, expression analysis by Western Blot suggested higher abundance in metacyclics [73], while proteomic studies revealed LmjF.02.0230 to be differentially expressed in amastigotes and LmjF.02.0190 to be constitutively expressed with higher peptide abundance in amastigotes [40], which is interesting because LPG is poorly – or not at all – expressed in this life stage. In both studies all the remaining genes of the array are predicted to be more abundant in promastigotes, which strengthens the argument of developmental regulation for functional differentiation within the tandem array and in this particular lineage.

The *Leishmania*-specific single-copy SCGL lineage likely arose from a transposition event from the SCGR array in chromosome 2 to chromosome 14. Members of this family are found in a paraphyletic clade with single gene copies in *Crithidia* and *Lotmaria*. When first described, LmjF.14.1400 was detected at low levels in all life cycle stages, compared to the high expression of SCGR and SCG members [73], which was corroborated by proteomics in *L. major* and *L. infantum*, where the gene was either not detected [74] or constitutively expressed at low abundance [40]. These data combined suggest that localization in the tandem array is essential for high protein expression and is yet another example of potential functional differentiation within the UGT family of Leishmaniinae.

Finally, the SCG lineage is *Leishmania-*specific whose genes are located at the telomeric regions of several chromosomes. In *L. major*, these genes have been shown to encode functional proteins, which are expressed in the parasite [73]. Most likely, the ancestor of this genus also possessed several copies of these UGTs, although their trace has been lost due to their highly unstable genomic location. Early investigation of developmental regulation revealed LmjF.07.1170 to be more abundant in promastigotes, but LmjF.31.3170 and LmjF.35.0010 in metacyclics and amastigotes [73]. However, this contrasts with proteomic studies in the same strain, which suggest higher protein abundance in amastigotes for all SCG genes [40]. Similarly, proteomic studies in *L. infantum* revealed differential expression at the amastigote level in 3 of the 4 homologs [74], and transcriptomic data for *L. mexicana* also suggest preferential expression in amastigotes [75]. Existing studies in *L. major* comparing metacyclic promastigotes to 4 and 24-h post-infection amastigotes also suggest gradual higher abundance in amastigotes [76]. The evidence for preferential expression in the intracellular amastigote stage is also consistent with the absence of these genes in monoxenic trypanosomatids. Recombination seems to be happening particularly between *L. major* sequences. Although evidence of positive selection to be acting upon this clade, as previously suggested [73], could not be found, it is possible that a combination of relaxation of negative selection and telomeric localization are aiding coincident evolution of SCG genes in most *Leishmania* species.

In summary, the scenario of UGT evolution in Leishmaniinae suggests the UGT repertoire of its ancestor was organised in a tandem array and that transpositions to telomeric regions of various chromosomes might have allowed parasite-specific expansion of the repertoire. This expansion has since become concerted, as a result of the high rate of recombination possibly by telomeric exchange.

### African trypanosomes sub-telomeric UGTs may have expanded to increase numbers of functionally redundant isoforms

In African trypanosomes, UGT orthology is largely conserved throughout the lineages. However, extensive duplication occurred in both *T. brucei* and *T. congolense* at the subtelomeres (Fig. 4).

Lineages 3–6 are under strong purifying selection, which likely reflects functional differences (Fig. 4). In *T. brucei*, lineage 3 and 4 are preferentially expressed in the BSF. These lineages represent N-acetylglucosaminyltransferase I and II, respectively, which are involved in the N-glycans biosynthetic pathway [77, 78]. Gene knockout studies and functional in vitro assays have showed that N-acetylglucosaminyltransferase I transfers GlcNAc to the 3-arm of trimannosyl core of N-linked glycans, while N-acetylglucosaminyltransferase II performs the transfer to the 6-arm. Additionally, unlike its homolog in multicellular organisms, the latter was shown not to be essential for parasite survival, although mutants do show branched instead of linear poly-N-acetyllactosamine chains at the arms of the trimannosyl core [78], suggesting at least some degree of redundancy or compensation mechanism. Being highly divergent from all other eukaryotic homologs, these sequences represent a parasite innovation through the adaptation of UGT family members to perform the N-acetylglucosaminyltransferase role of catalyzing ß1–2 glycosidic linkages. This is further emphasized by the report of a separate study suggesting that African trypanosomes UDP-sugar-dependent GT all belong to a single family evolved from a common ancestor of the ß3-glycosyltransferase [79], but have the ability to catalyse distinct linkages to account for the parasite extensive glycoconjugate repertoire.

The only single-copy lineage with evidence for preferential expression in the procyclic form is lineage 5 (Fig. 4). This lineage is under negative selection in all three African trypanosomes, suggesting a non-redundant function. The *T. brucei* homolog, Tb927.10.12290, has previously been functional characterized in a study that showed PCF mutants have smaller procyclins, resulting from modified GPI-anchor side chains, and thus suggesting Tb927.10.12290 to encode a GPI side-chain UDP-GlcNAc: βGal β1–3 GlcNAc- transferase [29]. Furthermore, the authors also suggested involvement in the N-linked poly LacNAc chain synthesis in BSF. The latter is interesting since it would explain why this gene has been conserved in *T. vivax* and potentially even duplicated to TvY486_0038690, since this parasite does not have a procyclic life stage. In the same study, this gene has also been linked to Tb927.2.3370 [29, 79]. Tb927.2.3370 has recently been functionally described through biochemical characterization of conditional null mutants under nonpermissive conditions [79]. This study revealed that the product of this gene is non-essential for the survival of *T. brucei* in culture, likely acting downstream the product of Tb927.10.12290, as a GPI side chain modifying UDP-Gal: βGlcNAc β1–3 Gal-transferase.

Apart from the link between Tb927.5.2760 and suramin efficacy and potential resistance together with 27 other genes (some of which shared N-acetylglucosamine biosynthesis activity [80]) not much is known about the function of lineage 6 (Fig. 4). An early study suggested that distinct COOH-termini in VSG impose distinct steric constrains on GPI-modifying galactosyltransferases activity [13]. Functional characterization of Tb927.5.2760, or its homologs in *T. congolense* and *T. vivax*, is required to investigate the affinity of these transferases for the distinct steric conformations, if any, displayed by the different VSG families of African trypanosomes.

Lineages 1 and 7 have greatly expanded in *T. brucei* and *T. congolense* and most genes are located in the sub-telomeres (Fig. 4). Subtelomeres are unstable genomic locations, where genes under neutral evolution may be transposed or expressed due to their proximity to other genes under positive selection. This may explain the unusual branch lengths in lineage 7, particularly in *T. brucei*, even though these genes rarely recombine nor are under positive selection. Eight of seventeen *T. brucei* genes of lineage 7 are preferentially expressed in the procyclic stage at the proteomic level, which would explain the absence of this particularly lineage in *T. vivax*, since this parasite does not have a fly midgut stage. Time-point proteomics analysis of stumpy to procyclic form differentiation identified five genes (Tb927.4.5260, Tb927.4.5270, Tb927.4.5280, Tb927.4.5290, and Tb11.v5.0880) in this clade to be up regulated only 12 h after differentiation induction, continuing until established procyclic stage [81]. This suggests these genes are involved in the late stages of stumpy form to procyclic differentiation. What remains to be explained is why *T. brucei* retains so many procyclic-specific UGTs, and whether members of this lineage are all non-essential or redundant as Tb927.2.3370. If so, it could be hypothesized that the family expansion and its subtelomeric localization may be advantageous for expression. In lineage 1, *T. brucei* and *T. vivax* genes seem to be constitutively expressed, but the lack of gene expansion in *T. vivax* is intriguing. This might be explained by crucial differences in parasite surface coating, such as a lower requirement of GPI-anchored proteins to be secreted.

### UGT expansion in Leishmaniinae and *Trypanosoma* occurred independently

To accommodate the requirements of a parasitic life cycle, both African trypanosomes and Leishmaniinae had to develop survival strategies in the shape of developmental regulation of protein expression. In this paper, we showed that UGTs have greatly expanded and are strongly developmentally regulated in both species. However, the specific characteristics of each life cycle have led to distinct approaches to the same challenge. To succeed in their obligate extracellular life cycle, African trypanosomes have developed mechanisms through which the parasite cell is covered by glycosylated proteins, e.g. procyclin or VSGs, which may account to up to 20% of the total protein in the cell [82]. To synthesize such enormous quantity of post-translationally modified proteins, the parasites require high dosage of UGTs to catalyse the various steps of GPI-anchor production and side chain glycosylation [13, 29, 79, 83]. Therefore, we propose that UGT sequences have moved to the subtelomeres to expand as functionally redundant isoforms. On the other hand, Leishmaniinae parasites are mostly intracellular and thus their survival must rely on defined developmentally regulated mechanisms that allow successful stage-specific adhesion and effective cell invasion, rather than protein abundance. In these parasites, UGTs catalyse the modification of phosphoglycan repeats in LPG and other surface (and secreted) glycoconjugates, whose defined combinations ensure transmissibility in the sand-fly vector and parasite fitness in the mammalian host [48]. Therefore, UGTs in Leishmaniinae have evolved under strong purifying selection, characterized by infrequent duplication, orthology retention, and lack of recombination. Together, these phenomena have resulted in the conservation of three functionally distinct sub-families, SCG, SCGR, and SCGL, comprised mostly of non-redundant enzymes.

## Conclusion

The UGT phylogeny shows that *Trypanosoma* and Leishmaniinae have diversified their UGT repertoires, relative to their free-living ancestor, which had considerably fewer UGT genes. The lineage we have discovered in *B. saltans* and *T. cruzi* may represent a remnant of this ancestral repertoire, and functional comparison of this lineage with parasite-specific UGT will be important in elucidating the precise benefit conferred on the ancestral parasites by these innovations. At present, gene expression profiles indicate that UGT genes diversified for similar reasons in both *Trypanosoma* and Leishmaniinae, i.e. to enable developmental regulation of UGTs that, like other functions, is necessary during multi-host life cycles. However, while these expansions may be responses to a common need, we have shown conclusively that they occurred independently. This supports the general hypothesis that dixenic life cycles in *Trypanosoma* and *Leishmania* evolved in parallel from different invertebrate-parasitic ancestors [84]. Among Leishmaniinae, strong purifying selection of UGT sequences, their infrequent duplication and lack of recombination indicate that diversification occurred to provide functionally distinct and non-redundant enzymes, essential for parasite transmission through the fly host. Conversely, neutral evolution of African trypanosome UGT sequences and their frequent and relatively recent duplication in sub-telomeric regions, suggests that expansion serves to increase gene dosage of functionally redundant isoforms. Thus, the circumstances of UGT genes in *Trypanosoma* and Leishmaniinae betray how these two lineages evolved a similar solution to independently meet their superficially common need to decorate their cell surfaces for infection and transmission. In this way, the UGT phylogeny is consistent with the evolutions of the cell-surface proteins that they decorate, which have also evolved independently. This independence only reinforces the importance of cell-surface interactions in determining parasite fitness, and shaping their genomes.

## Additional files

Additional file 1: **Figure S1.** Conserved genomic locus in *L. major* Friedlin, *L. infantum* JPCM5, *L. braziliensis* M2904, and *Crithidia fasciculata* CfC1. The UDP-glycosyltransferase genes are shaded yellow, flanking genes are shaded orange; sequence homology is illustrated by gray vertical bars. Gene terminology is according to Tritrypdb identifiers. Comparisons were obtained with Artemis Comparison Tool (ACT) [85]. (PNG 886 kb)

Additional file 2: **Figure S2.** Subtelomeric genomic locus in chromosome 25 of *L. major* Friedlin, *L. infantum* JPCM5, *L. mexicana* U1103, and *L. braziliensis* M2904. The UDP-glycosyltransferase genes are shaded yellow, flanking genes are shaded orange; sequence homology is illustrated by gray vertical bars. Gene terminology is according to Tritrypdb identifiers. Comparisons were obtained with Artemis Comparison Tool (ACT) [85], (PNG 357 kb)

Additional file 3: **Table S1.** Results of selection tests on *Leishmania*. PARRIS [65] searches for evidence of positive selection at individual sites. PAML [67] searches for positive selected sites. REL [65], SLAC [64], FEL [64], and FUBAR [66] search for evidence of positive and negative selection based on dN/dS ratios. (DOCX 27 kb)
