# Supplementary figures and images for: UDP-glycosyltransferase genes in trypanosomatid genomes have diversified independently to meet the distinct developmental needs of parasite adaptations
Source: BMC Evol Biol. 2018 Mar 14;18:31. doi: 10.1186/s12862-018-1149-6 (PMC5853035; doi:10.1186/s12862-018-1149-6)

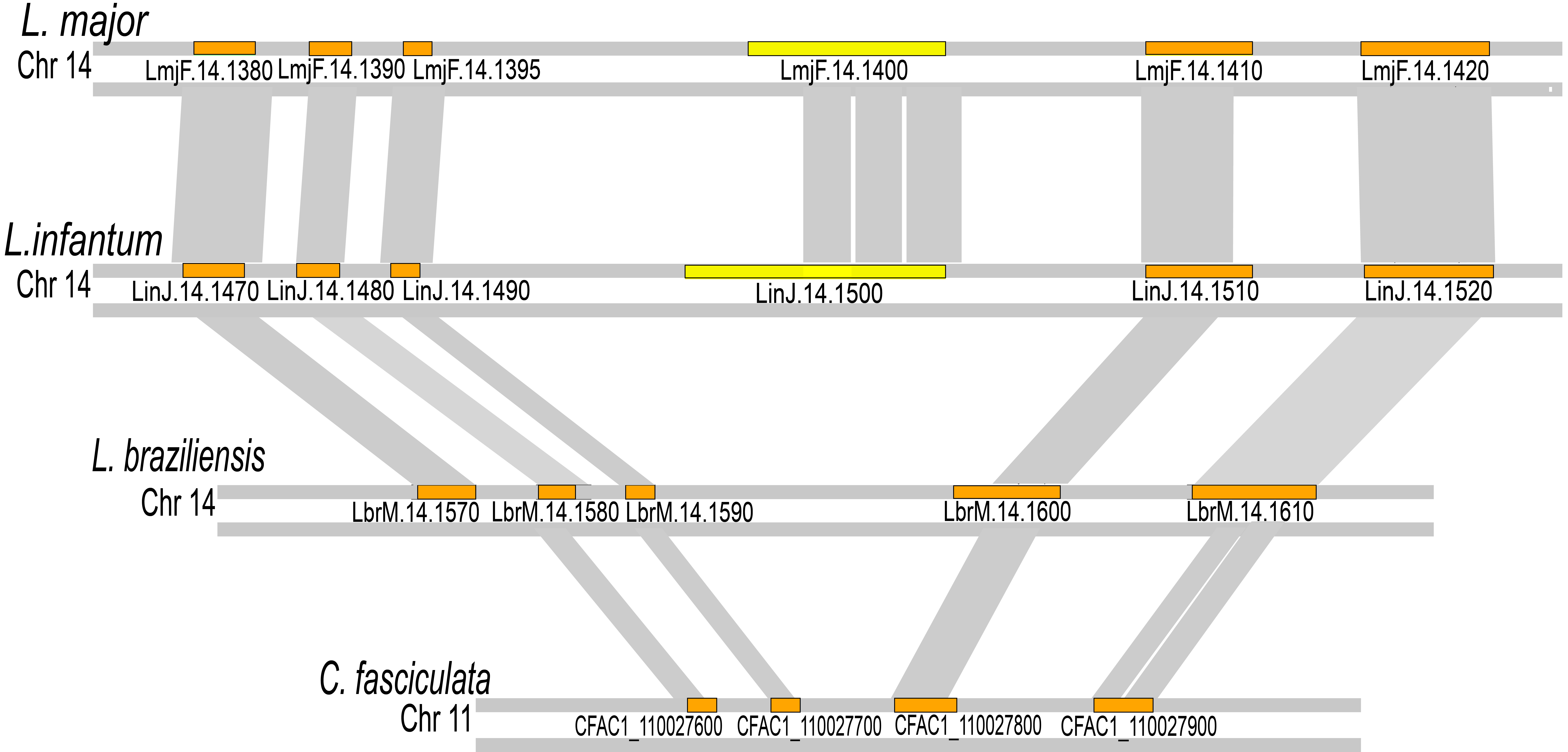

Supplement: Supplementary file 1 — Figure S1. Conserved genomic locus in L. major Friedlin, L. infantum JPCM5, L. braziliensis M2904, and Crithidia fasciculata CfC1. The UDP-glycosyltransferase genes are shaded yellow, flanking genes are shaded orange; sequence homology is illustrated by gray vertical bars. Gene terminology is according to Tritrypdb identifiers. Comparisons were obtained with Artemis Comparison Tool (ACT) [85]. (PNG 886 kb) [file 12862_2018_1149_MOESM1_ESM.png]

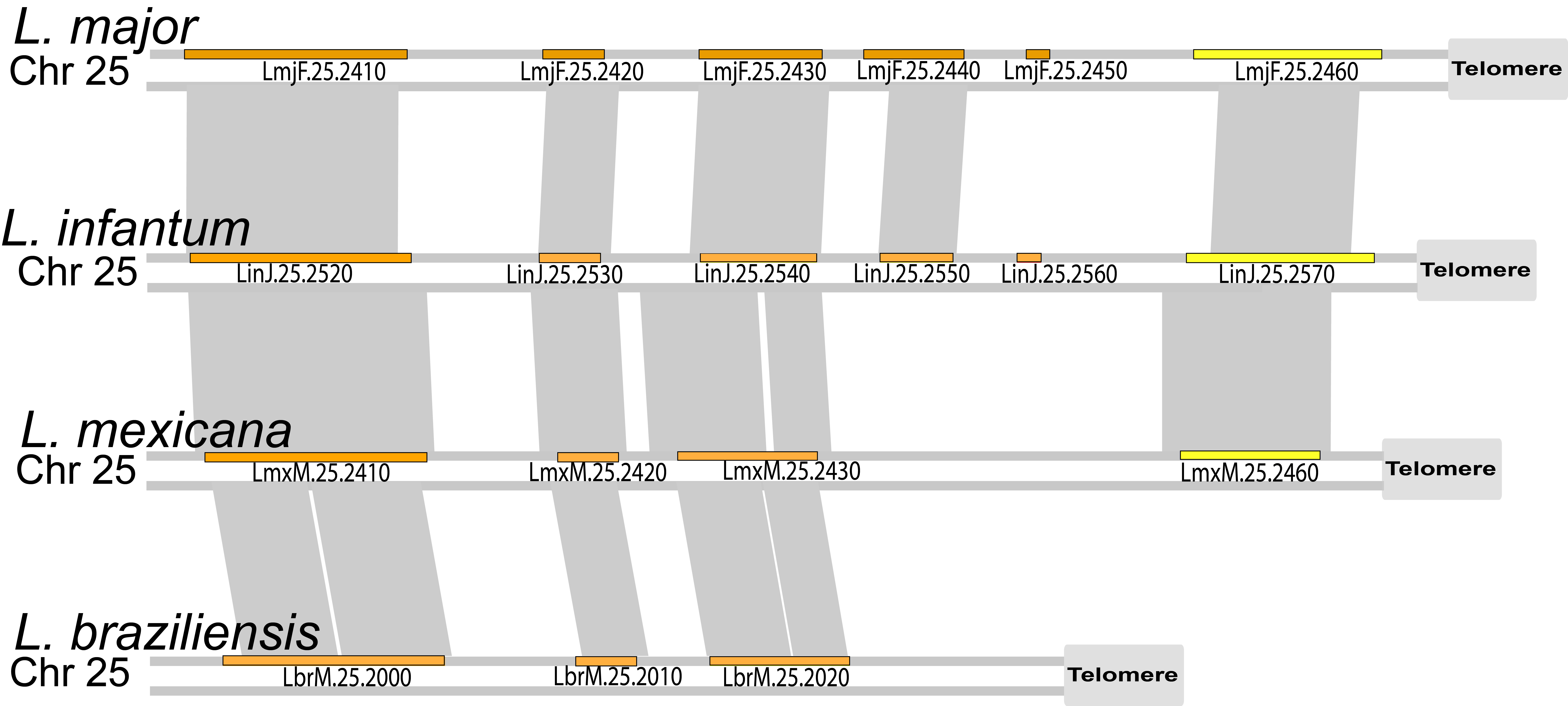

Supplement: Supplementary file 2 — Figure S2. Subtelomeric genomic locus in chromosome 25 of L. major Friedlin, L. infantum JPCM5, L. mexicana U1103, and L. braziliensis M2904. The UDP-glycosyltransferase genes are shaded yellow, flanking genes are shaded orange; sequence homology is illustrated by gray vertical bars. Gene terminology is according to Tritrypdb identifiers. Comparisons were obtained with Artemis Comparison Tool (ACT) [85], (PNG 357 kb) [file 12862_2018_1149_MOESM2_ESM.png]
