# Supplementary material for: UDP-glycosyltransferase genes in trypanosomatid genomes have diversified independently to meet the distinct developmental needs of parasite adaptations
Source: BMC Evol Biol. 2018 Mar 14;18:31. doi: 10.1186/s12862-018-1149-6 (PMC5853035; doi:10.1186/s12862-018-1149-6)
Supplement: Supplementary file 3 — Table S1. Results of selection tests on Leishmania. PARRIS [65] searches for evidence of positive selection at individual sites. PAML [67] searches for positive selected sites. REL [65], SLAC [64], FEL [64], and FUBAR [66] search for evidence of positive and negative selection based on dN/dS ratios. (DOCX 27 kb) [file 12862_2018_1149_MOESM3_ESM.docx]

Additional file Table S1 Results of selection tests on *Leishmania*. PARRIS [60] searches for evidence of positive selection at individual sites. PAML [62] searches for positive selected sites. REL [60], SLAC [59], FEL [59], and FUBAR [61] search for evidence of positive and negative selection based on dN/dS ratios.

| Region | PARRIS | PAML | REL | SLAC | FEL | FUBAR |
| --- | --- | --- | --- | --- | --- | --- |
| *L. infantum* SCG | No | 34+ | 131+ 1- | 0 | 1+ 11- | 10- |
| *L. infantum* -ve | No | 37+ | 0 | 1- | 4+ 3- | 1- |
| *L. infantum* SCGR | No | 24+ | 3+ 14- | 1- | 1+ 5- | 1- |
| *L. major* SCG | No | 4+ | 29- | 0 | 11- | 7+ 11- |
| *L. major* -ve | No | 32+ | 0 | 1- | 3+ 3- | 1- |
| *L. major* SCGR | No | 15+ | 13+ 357- | 0 | 44- | 4+ 96- |
| *L. mexicana SGC* | No | 9to20+ | 0 | 0 | 18- | 14- |
